# Four-class ASME BCI: investigation of the feasibility and comparison of two strategies for multiclassing

**DOI:** 10.3389/fnhum.2024.1461960

**Published:** 2024-11-26

**Authors:** Simon Kojima, Shin'ichiro Kanoh

**Affiliations:** ^1^Graduate School of Engineering and Science, Shibaura Institute of Technology, Tokyo, Japan; ^2^College of Engineering, Shibaura Institute of Technology, Tokyo, Japan

**Keywords:** brain-computer interface, electroencephalogram, event-related potential, auditory scene analysis, stream segregation, machine learning, NASA-TLX

## Abstract

**Introduction:**

The ASME (stands for Auditory Stream segregation Multiclass ERP) paradigm is proposed and used for an auditory brain-computer interface (BCI). In this paradigm, a sequence of sounds that are perceived as multiple auditory streams are presented simultaneously, and each stream is an oddball sequence. The users are requested to focus selectively on deviant stimuli in one of the streams, and the target of the user attention is detected by decoding event-related potentials (ERPs). To achieve multiclass ASME BCI, the number of streams must be increased. However, increasing the number of streams is not easy because of a person's limited audible frequency range. One method to achieve multiclass ASME with a limited number of streams is to increase the target stimuli in a single stream.

**Methods:**

Two approaches for the ASME paradigm, ASME-4stream (four streams with a single target stimulus in each stream) and ASME-2stream (two streams with two target stimuli in each stream) were investigated. Fifteen healthy subjects with no neurological disorders participated in this study. An electroencephalogram was acquired, and ERPs were analyzed. The binary classification and BCI simulation (detecting the target class of the trial out of four) were conducted with the help of linear discriminant analysis, and its performance was evaluated offline. Its usability and workload were also evaluated using a questionnaire.

**Results:**

Discriminative ERPs were elicited in both paradigms. The average accuracies of the BCI simulations were 0.83 (ASME-4stream) and 0.86 (ASME-2stream). In the ASME-2stream paradigm, the latency and the amplitude of P300 were shorter and larger, the average binary classification accuracy was higher, and the average weighted workload was smaller.

**Discussion:**

Both four-class ASME paradigms achieved a sufficiently high accuracy (over 80%). The shorter latency and larger amplitude of P300 and the smaller workload indicated that subjects could perform the task confidently and had high usability in ASME-2stream compared to ASME-4stream paradigm. A paradigm with multiple target stimuli in a single stream could create a multiclass ASME BCI with limited streams while maintaining task difficulty. These findings expand the potential for an ASME BCI multiclass extension, offering practical auditory BCI choices for users.

## 1 Introduction

The brain-computer interface (BCI) allows users to control external devices without muscle activation by decoding their intention from neural signals. Numerous studies have been conducted on BCI for patients who have severe motor impairments, completely locked-in syndrome (CLIS), amyotrophic lateral sclerosis (ALS) or spinal cord injury (SCI) (Wolpaw et al., 2000; King et al., 2013; Guger et al., 2017; Zhang et al., 2017; Guger et al., 2020) and for healthy people (Holzner et al., 2009; Kosmyna et al., 2016; Yu et al., 2016; Pan et al., 2017; Gao et al., 2018; Park et al., 2019). Electroencephalogram (EEG) is widely used to measure brain activity because they are suitable for measuring signals with high temporal resolution via portable devices (Vidal, 1973; Wolpaw et al., 2002; Rao, 2019).

Among EEG-based BCIs, synchronous BCIs detect a stereotypical brain response generated after the subject is presented with a stimulus (Rao, 2019). Some synchronous BCIs that use visual stimuli have been proposed. These visual BCIs have performed well (Cheng et al., 2002; Gao et al., 2003; Bin et al., 2009; Thielen et al., 2021; Martínez-Cagigal et al., 2021). However, BCIs based on visual stimuli occupy the user's sight, and thus, cannot be used by patients with visual impairments.

In contrast, auditory BCIs, which use auditory stimuli, do not require visual modality and can be used without restricting the user's visual functions. Hill et al. proposed a famous example of an early auditory BCI (Hill et al., 2004). They presented two different oddball sequences to the right and left ears of the subject. In an oddball paradigm, subjects are presented with a sequence of frequent tones (i.e., standard or nontarget stimuli). In contrast, some are infrequently replaced with different stimuli (i.e., deviant or target stimuli). When a listener focuses on a sequence (e.g., by counting the number of target stimuli), event-related potentials, such as P300 and N200, are elicited by the target stimulus presented (Luck, 2014). In their system, the direction of the subject's focus was estimated by detecting ERP responses to the target stimuli using a support vector machine (SVM). Furdea et al. (2009) proposed the auditory speller BCI. In this system, a five-by-five matrix consisting of alphabet letters was coded with acoustically presented numbers. The subjects selected the target character by focusing selectively on the sound stimuli corresponding to the row and column numbers in sequence. This system was also tested on locked-in patients (Kübler et al., 2009). Another system was proposed by Schreuder et al. (2010). The subjects were surrounded by eight loudspeakers at the ear level, and the target direction of the user's attention was detected. Musso et al. (2022) proposed a BCI-based language rehabilitation system for poststroke aphasia patients with this paradigm. Höhne et al. (2011) proposed a 9-class auditory BCI by presenting sound stimuli with three pitches from three directions. The target direction and pitch were detected using Fisher discriminant analysis (FDA).

Despite many proposed auditory BCIs, their performance is lower than that of visual-based BCIs (Furdea et al., 2009; Belitski et al., 2011; Oralhan, 2019). However, patients with late-stage ALS are known to have unreliable gaze control (Choi et al., 2023); thus, proposing systems that are not dependent on visual modalities is crucial for these patients.

Furthermore, this approach may not be the most suitable choice for some users since it occupies user vision, which is a sensory modality frequently used in daily life. Therefore, developing a high-performance and practical auditory BCI is important.

The auditory BCIs mentioned above use relatively simple paradigms, such as distinguishing between high- and low-pitched tones or selecting one sound from spatially located sounds. However, the human auditory system is capable of very complex processing and can highly discriminate between various sounds, and high-performance auditory BCIs may be achieved if we can fully utilize human auditory abilities. Thus, an auditory BCI based on stream segregation was proposed (Kanoh et al., 2008, 2010; Kojima and Kanoh, 2024). Hereinafter, this paradigm is referred to as the auditory stream segregation, Multiclass, ERP (ASME). Stream segregation is an auditory illusion that makes perceiving alternately presented sounds as segregated multiple sound streams possible (Bregman, 1990; Shamma and Micheyl, 2010; Snyder and Alain, 2007). In the original work (Kanoh et al., 2008, 2010), two different oddball sequences with different pitches were presented simultaneously. The user's target stream was detected by linear discriminant analysis (LDA), which is a two-class system. The sound sequence was presented only to the subject's ear. The detection accuracy reached 95%, and the information transfer rate (ITR) was approximately 5 bits/min. This work was novel compared to that of conventional auditory BCIs in terms of its utilization of human auditory ability. In addition, a multiclass auditory BCI can be created with monaural channel sounds, and it can be used by a user who has an impairment in one ear.

**Figure 1:**
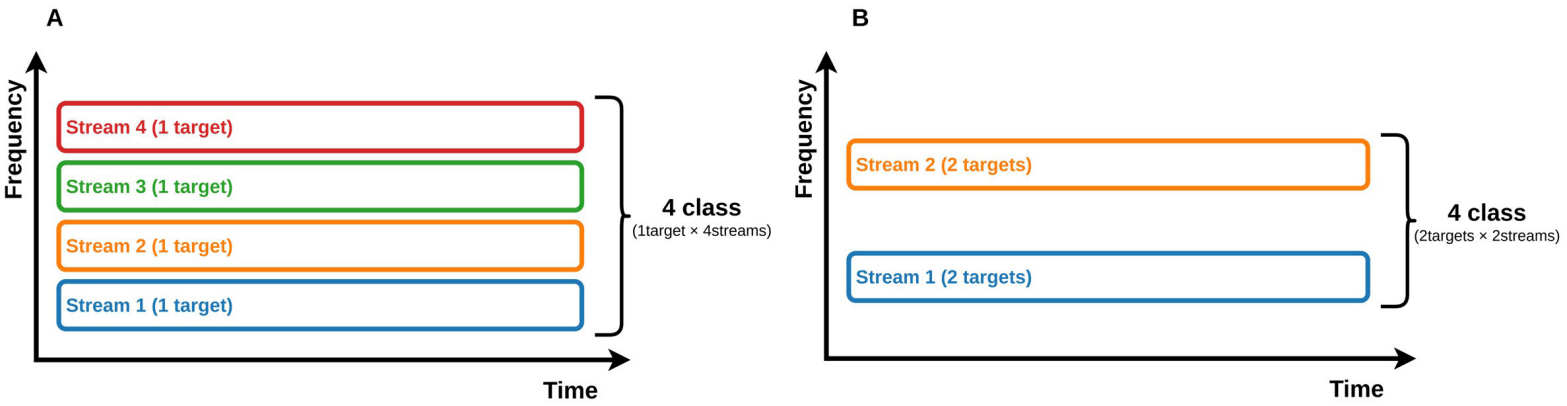
The concept of an ASME with multiple target stimuli in each stream. **(A)** A four-class ASME paradigm with four streams in which each stream has one target stimulus. **(B)** A four-class ASME paradigm with two streams in which each stream has two target stimuli.

**Figure 2:**
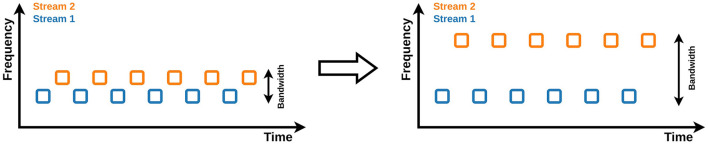
The influence of spacing in frequency between streams on stream segregation and bandwidth of stimuli. When the spacing between streams is wide, perceiving the sequence as segregated multiple streams is easier; however, the bandwidth of the stimuli becomes broader.

We proposed a three-class ASME paradigm (Kojima and Kanoh, 2024). However, to realize a four-class ASME paradigm, the simplest solution is to increase the number of streams, i.e., to present four streams, each of which is a classic two-stimulus oddball sequence (see Figure 1A). However, in the ASME paradigm, as the number of streams increases, it becomes difficult to perceive the sequence as segregated streams. The spacing in frequency between streams needs to be wider to make the sequence easier to perceive as segregated streams (Bregman, 1990); however, a wider spacing in frequency widens the stimuli bandwidth (see Figure 2). Since the human audible range is limited to 20 − 20, 000Hz (Rosen and Howell, 2011), the maximum number of streams is limited. One method to increase the number of selections without increasing the number of streams is to include multiple target stimuli in a single oddball sequence. Halder et al. (2010) tested the three-stimulus auditory oddball sequence and achieved high accuracy and ITR. The three-stimulus oddball has two different target stimuli. The four-class ASME paradigm can be achieved with two streams utilizing the three-stimulus oddball (see Figure 1B). Thus, we propose two different strategies for realizing the four-class ASME paradigm: (1) presenting four streams, each of which is a two-stimulus oddball (Figure 1A), and (2) presenting two streams, each of which is a three-stimulus oddball (Figure 1B).

Thus, in this study, two different topics were investigated: (1) the feasibility of the four-class ASME BCI with each strategy and (2) comparing two strategies for the four-class ASME paradigm. The ERP responses were evaluated, and the feasibility of the BCI application was tested by offline analysis and simulation. Additionally, the usability of each paradigm was evaluated by the NASA-TLX (Hart and Staveland, 1988; Hart, 2006; Ortega-Gijon and Mezura-Godoy, 2019).

## 2 Materials and methods

### 2.1 Stimuli

In this section, two ASME paradigms, (a) four-stream paradigms with two oddball stimuli and (b) two-stream paradigms with three oddball stimuli, are described.

#### 2.1.1 (a) ASME consisting of four streams with a two-stimuli oddball (ASME-4stream)

**Figure 3:**
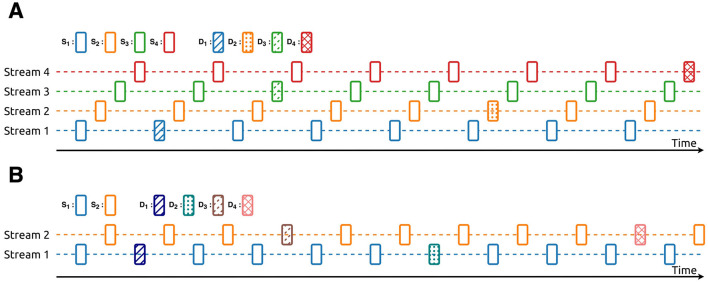
The sequences used in the experiment. **(A)** The sequence used in the ASME-4stream paradigm. **(B)** The sequence used in the ASME-2stream paradigm.

Figure 3A shows the sequence for the ASME paradigm, which has four streams with two-stimuli oddballs (ASME-4stream paradigm). *S*_*n*_ are standard stimuli, and *D*_*n*_ are deviant stimuli in stream *n*. Table 1 shows the frequency of each stimulus. The stimulus onset asynchrony (SOA) was set to 0.15*s*. In one trial, 600 stimuli were presented, and the duration was approximately 90 s. In each stream, the presentation ratio was *S*_*n*_:*D*_*n*_ = 9:1.

**Table 1:** Frequencies for stimuli used for ASME-4stream paradigm.

**Stream**	**Stimulus**	**Frequency (Hz)**
Stream 1	*S*_1_	110.00
	*D*_1_	123.47
Stream 2	*S*_2_	415.30
	*D*_1_	466.16
Stream 3	*S*_3_	1,567.98
	*D*_3_	1,760.00
Stream 4	*S*_4_	5,919.91
	*D*_4_	6,644.86

#### 2.1.2 (b) ASME consisting of two streams with a three-stimuli oddball (ASME-2stream)

Figure 3B shows the sequence for the ASME paradigm, which has four streams with a two-stimuli oddball (ASME-2stream paradigm). *S*_*n*_ are standard stimuli, and *D*_*n*_ are deviant stimuli. Table 2 shows the frequency of each stimulus. In this paradigm, each stream had two different deviant stimuli: one had a lower frequency, and the other had a higher frequency. The SOA was set to 0.3 s. In one trial, 300 stimuli were presented, and the duration was approximately 90 s. In each stream, the presentation ratio was *S*_*n*_:*D*_*L*_:*D*_*H*_ = 8:1:1, where *D*_*L*_ and *D*_*H*_ are the number of deviant stimuli that had lower and higher frequencies, respectively.

**Table 2:** Frequencies for the stimuli used for the ASME-2stream paradigm.

**Stream**	**Stimulus**	**Frequency (Hz)**
Stream 1	*S*_1_	523.3
	*D*_1_	440.0
	*D*_2_	622.3
Stream 2	*S*_2_	3,136.0
	*D*_3_	2,637.0
	*D*_4_	3,729.3

#### 2.1.3 Differences in SOA and presentation ratio between the two conditions

In the ASME-4stream and ASME-2stream conditions, the SOA and presentation ratio were different.

The difference in SOA was made to keep the SOA of the stimulus sequence within the attended stream consistent when selective attention was directed to a specific stream while ignoring others. In a sequence with N streams and an overall SOA of t seconds, the within stream SOA_stm_ becomes *N*×*t*. In both the ASME-4stream and ASME-2stream conditions, the SOA_stm_ was set to 0.6 seconds. Additionally, It has been shown that changes in SOA_stm_ can influence the ease of stream segregation perception (Bregman, 1990), and the SOA_stm_ used in this study was chosen to ensure that each sound stream could be sufficiently perceived as a segregated stream.

The difference in presentation ratio was made to keep the presentation ratio of the target and nontarget stimuli equal when selective attention was paid to a specific deviant stimulus. In the ASME-2stream condition, two deviant stimuli are embedded within each stream. When attention is paid to one of the deviant stimuli, the attended stimulus becomes the target stimulus, and the other stimuli can be treated as nontarget stimuli. For example, in the ASME-2stream condition, the presentation ratio was *S*:*D*_1_:*D*_2_ = 8:1:1, however when attention is paid to either *D*_1_ or *D*_2_, the ratio between the target (*T*) and nontarget stimuli (*nT*) became *nT*:*T* = 1:9, which was the same as in the ASME-4stream condition.

### 2.2 Experimental design

**Figure 4:**
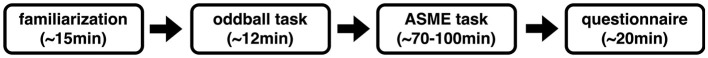
The experimental design.

Figure 4 shows the structure of an experiment. In the familiarization session, all subjects were presented with both the ASME-4stream and ASME-2stream paradigms. Next, the simple auditory oddball paradigm was conducted with the following parameters in the oddball session. The frequencies of standard and deviant stimuli were 500Hz and 1, 000Hz, respectively. The SOA was set to 1.0 s, and the presentation ratio was *S*:*D* = 5:1, where *S* and *D* were the number of standard and deviant stimuli, respectively. In the ASME task session, 12 runs of the ASME-2stream and ASME-4stream paradigms were conducted alternately. In a single run, four trials were conducted. Before starting each trial, a screen was placed in front of the subjects and they were instructed on which stream and stimuli to focus on. After the instruction, each standard stimulus of all streams was presented for one second to provide pitch information for the subjects to target the streams better. During the trial, subjects were requested to focus on and count the target deviant stimuli in the target stream.

After the ASME task session, the NASA-TLX questionnaire was administered to score the subjective mental workload for each task. Since all subjects spoke Japanese, the translated NASA-TLX was used (Haga and Mizukami, 1996). With the NASA-TLX, the following six indexes were scored: mental demand, physical demand, temporal demand, performance, effort, and frustration, and the weighted workload (WWL) was obtained.

### 2.3 Subjects

Fifteen subjects (aged between 21 and 24 years, mean = 22.8 years, two females) were recruited for this study. The study protocol was approved by the Review Board on Bioengineering Research Ethics of the Shibaura Institute of Technology and was conducted in accordance with the Declaration of Helsinki. Before the experiment, the subjects were given information orally and in writing. Written informed consent was obtained from all the subjects. No subject had known cranial nerve diseases or hearing problems.

### 2.4 EEG and EOG measurement

Sixty-four-channel electroencephalogram (EEG) (Fp1, Fp2, AF7, AF3, AFz, AF4, AF8, F7, F5, F3, F1, Fz, F2, F4, F6, F8, FT9, FT7, FC5, FC3, FC1, FCz, FC2, FC4, FC6, FT8, FT10, T7, C5, C3, C1, Cz, C2, C4, C6, T8, TP9, TP7, CP5, CP3, CP1, CPz, CP2, CP4, CP6, TP8, TP10, P7, P5, P3, P1, Pz, P2, P4, P6, P8, PO7, PO3, POz, PO4, PO8, O1, Oz, and O2) and two-channel (vertical and horizontal) electrooculogram (EOG) were measured by BrainAmp (Brain Products, Germany) at a 1, 000Hz sampling frequency with passive Ag/AgCl electrodes (EasyCap, Germany). The electrodes were placed according to the extended 10-20 system. The reference and ground electrodes were placed on the right and left mastoid, respectively. The subjects sat on a comfortable chair in a soundproofing electromagnetically shielded room.

### 2.5 EOG artifact removal

EOG artifact removal was conducted as follows. First, the recorded EEG data from each run were highpass filtered by a zero-phase 2nd-order Butterworth filter with a cut-off frequency of 1.0 Hz to remove slow drift and were concatenated along the time domain.

After filtering, principal component analysis (PCA) was applied to the EEG data, and 15 principal components (PCs) were selected.

Next, independent component analysis (ICA) with the FastICA algorithm was applied to the 15 PCs. Two channels of EOG data (vertical and horizontal) were bandpass filtered by a 2nd-order Butterworth filter in the range of 1–10 Hz. The Pearson correlation coefficient between each IC and EOG channel was calculated. The IC, which had the highest correlation with the vertical and horizontal EOG, respectively, was set to zero.

Before applying ICA, 15 components were selected using PCA, as it is known that dimensionality reduction with PCA can improve the quality of the artifact separation (Winkler et al., 2015; Hyvarinen et al., 2001).

### 2.6 ERP analyses

All measured data after EOG artifact removal were bandpass filtered by a zero-phase 2nd order Butterworth filter in the range of 1–40 Hz. Responses to each stimulus were epoched in the range from −0.1s to 1.2s relative to stimulus onset. Then, all epochs were downsampled to 250Hz. Signed-*r*^2^ values (Blankertz et al., 2011) were obtained to visualize the separability between the responses to the target and nontarget stimuli. The target and nontarget stimuli were defined as follows. All analyses were performed using Python 3.8.18 and MNE-python 1.5.0 (Gramfort et al., 2013).

- ASME-4stream

The deviant stimuli in the target stream were the target, and the standard stimuli in the target stream, and all stimuli in the nontarget stream were nontarget. For example, when Stream 1 was the target stream, *D*_1_ was the target, and {*S*_1_, *S*_2_, *S*_3_, *S*_4_, *D*_2_, *D*_3_, *D*_4_} was the nontarget.

- ASME-2stream

The attended deviant stimuli in the target stream were the target, and the unattended deviant and standard stimuli in the target stream and all stimuli in the nontarget stream were nontarget. For example, when the target stream was Stream 1 and *D*_1_ was attended, *D*_1_ was the target and {*S*_1_, *S*_2_, *D*_2_, *D*_3_, *D*_4_} was the nontarget.

The onset and peak amplitude of P300 responses were estimated with a bootstrap procedure. For this analysis, the following five EEG channels (Fz, FCz, Cz, FPz, and Pz) were used, as it is known that P300 has peak amplitude on these channels (Polich, 2007).

**(1) P300 peak amplitude**

1. The 80% of the samples of the responses to the target stimuli were taken randomly and averaged across epochs.
2. The peak amplitude in the range of 0.2–0.5 seconds was obtained as *v*_*i*_.
3. Procedures 1 and 2 were repeated 1,200 times.
4. **v**∈**R**^1, 200^ was averaged and determined as the peak amplitude.

**(2) P300 onset latency**

1. The 80% of the samples of the responses to the target stimuli (*X*_*target*_) and standard stimuli (*X*_*standard*_) were taken randomly.
2. The time sample when the result of the one-sided Welch's t test between *X*_*target*_ and *X*_*standard*_ was significant (*p* < 0.05) for the first time and the corresponding time stamp was in between 0.2–0.5 seconds was taken as *t*_*i*_. If there was no significant difference, it was excluded from the subsequent analysis as a bootstrap sample in which P300 was not observed.
3. Procedures 1 and 2 were repeated 250 times.
4. $\text{t}\in {\text{R}}^{250-N_{f}}$ was averaged and determined as the P300 onset latency. *N*_*f*_ is the number of bootstrap samples for which no significant difference was observed in procedure 2.

### 2.7 Binary classification

After removing EOG artifacts using ICA, all measured data were bandpass filtered by a zero-phase 2nd-order Butterworth filter in the range of 0.1–8 Hz, and responses to each stimulus were epoched in the range of −0.1–1.2 s relative to stimulus onset. Then, all epochs were downsampled to 250 Hz. The mean amplitudes in the ten intervals (0.1 second, non-overlapping intervals from 0 to 1.0 seconds relative to the stimulus onset) were used as the classification feature. The dimensions of the feature vector were 10 intervals × 64 channels = 640. The classification accuracy (AUC: area under the receiver operating characteristic curve) between the responses to the target and nontarget stimuli was obtained by shrinkage linear discriminant analysis (shrinkage-LDA) (Blankertz et al., 2011) with 4-fold chronological cross-validation. For the binary classification, the chance level was 0.5. All analyses were performed with Python 3.8.18, scikit-learn 1.2.0 (Pedregosa et al., 2011). For shrinkage-LDA, implementation included in open source python package toeplitzlda (Sosulski and Tangermann, 2022) 0.2.6 was used.

### 2.8 BCI simulation (four-class classification)

In the BCI simulation, the target class of each trial out of four classes was estimated. Since six runs were measured for each paradigm, 3-fold chronological cross-validation (two runs for training the machine learning model and four runs for testing) was conducted. First, for training data from two runs, the unmixing and mixing matrix of ICA for removing EOG components was computed by the method described in Section 2.5. Then, the EOG artifact was removed from the training data, the feature vector was obtained using the same method described in Section 2.7, and shrinkage-LDA was trained. The classification output $f({\text{x}}_{i})={\text{w}}^{T}{\text{x}}_{i}+b$ was defined as follows, where ***x*_*i*_** is a feature vector, **w** is the weight vector obtained by LDA, and *b* is a bias. Each feature vector ***x*_*i*_** has a corresponding class label *y*_*i*_∈{−1, 1}, and it is assumed that class label +1 is the target and −1 is nontarget. The LDA was trained as *f*(**x**)≥0 if ***x*_*i*_** was in class +1 and *f*(**x**) < 0 if ***x*_*i*_** was in class −1. For the testing data of the four runs, the EOG components were zeroes by the unmixing and mixing matrix of ICA computed with the training data. The feature vectors for each trial were computed from the responses to all deviant stimuli in the trial. Then, the classifier output *f*(***x*_*i*_**) for each feature was computed, and the class with the largest mean value of classifier output was estimated as the final classification result. The classification results were evaluated for accuracy. For the BCI simulation, the theoretical chance level was 1/4 = 0.25. Due to the limited number of samples, the statistical significance of classification accuracy using a binomial cumulative distribution was also evaluated (Combrisson and Jerbi, 2015). Since we had 24 samples‘ for each condition, it was a 4-class classification, and the threshold for statistical significance of the accuracy *P*_*th*_ was *P*_*th*_ = 0.42 at *p* < 0.05. The detailed method and equations can be found in Combrisson and Jerbi (2015).

All analyses were performed with Python 3.8.18, scikit-learn 1.2.0 (Pedregosa et al., 2011). For shrinkage-LDA, implementation included in open source python package toeplitzlda (Sosulski and Tangermann, 2022) 0.2.6 was used.

The ITR is the amount of information communicated by a system per unit time (Rao, 2019). The ITR can be expressed as follows (Schreuder et al., 2010):


(1) $$\begin{array}{l} R=\log_{2}\left(N\right)+Plog_{2}\left(P\right)+\left(1-P\right)log_{2}(\frac{1-P}{N-1}) \end{array}$$

(2) $$\begin{array}{l} B=VR \end{array}$$

where *N* is the number of classes, *P* is the classification accuracy, *V* is the classification speed in trials/minute, *R* is the ITR in bits/trial, and *B* is the ITR in bits/minute.

## 3 Results

### 3.1 ERP analysis

**Figure 5:**
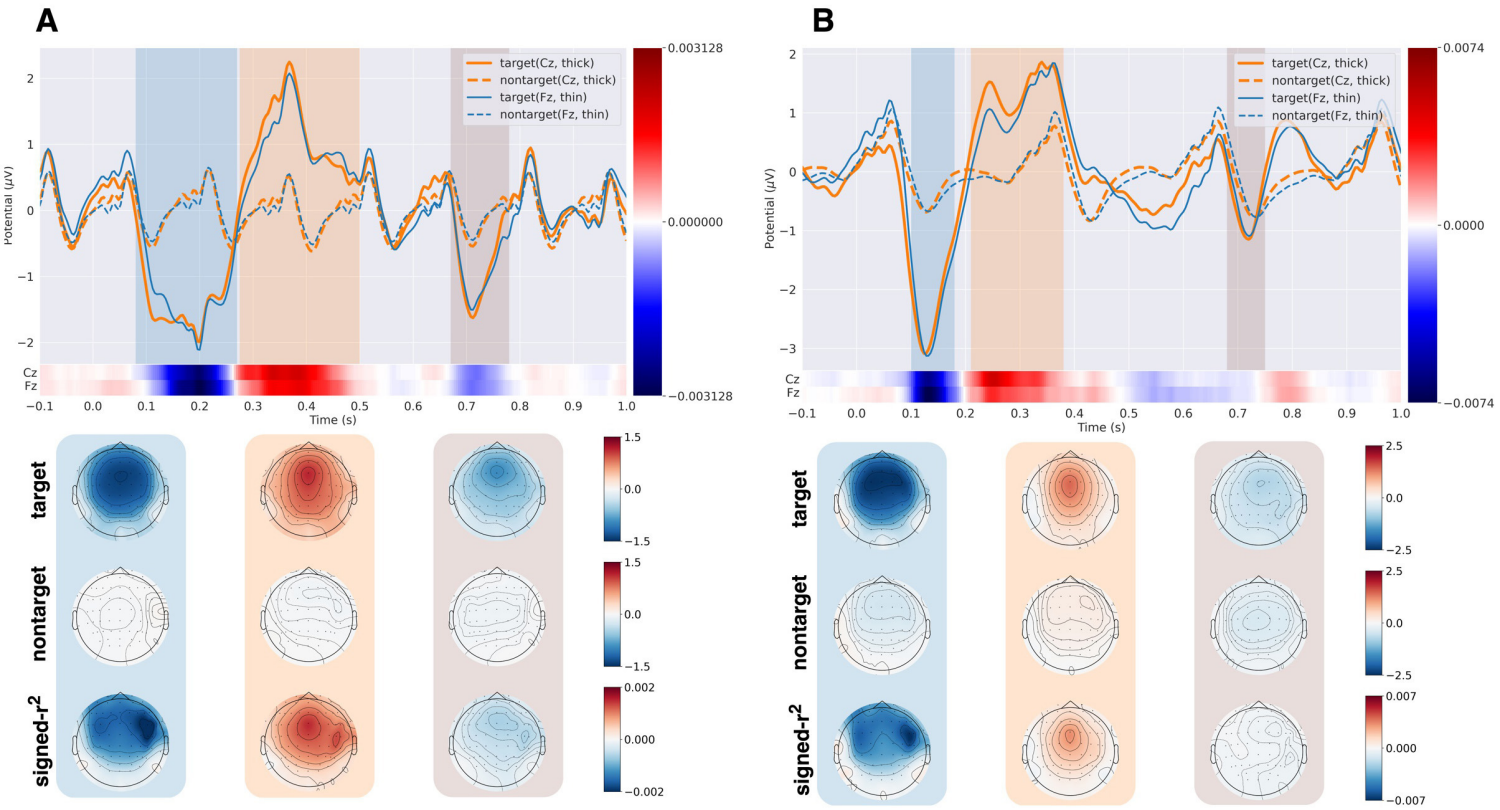
The grand average ERP responses in channels Cz and Fz for the ASME-4stream **(A)** and the ASME-2stream **(B)**. The colormap below the ERP plot shows the signed-r^2^ value at each time point. Each topography map shows the responses to the target, nontarget stimuli, and signed-*r*^2^ values. The time ranges used for the topography map are shown in the ERP plot in colored mesh.

Figure 5 shows the grand average ERP responses to target and nontarget stimuli and their topographic maps for the ASME-4stream and ASME-2stream paradigms, respectively. The signed-*r*^2^ value (Blankertz et al., 2011) between the responses to target and nontarget stimuli was also shown at the bottom of the ERP time courses. For both paradigms, N200 and P300 responses were elicited by the target stimuli. The peak amplitude of the N200 component was greater in the ASME-2stream paradigm, and the peak amplitude of the P300 component was greater in the ASME-4stream paradigm. In both paradigms, N700 responses were also elicited to the target stimuli, where the difference between target and nontarget stimuli is greater in the ASME-4stream paradigm. The N200 responses were elicited front-central in both paradigms. However, the discriminability was greater in the right hemisphere.

**Figure 6:**
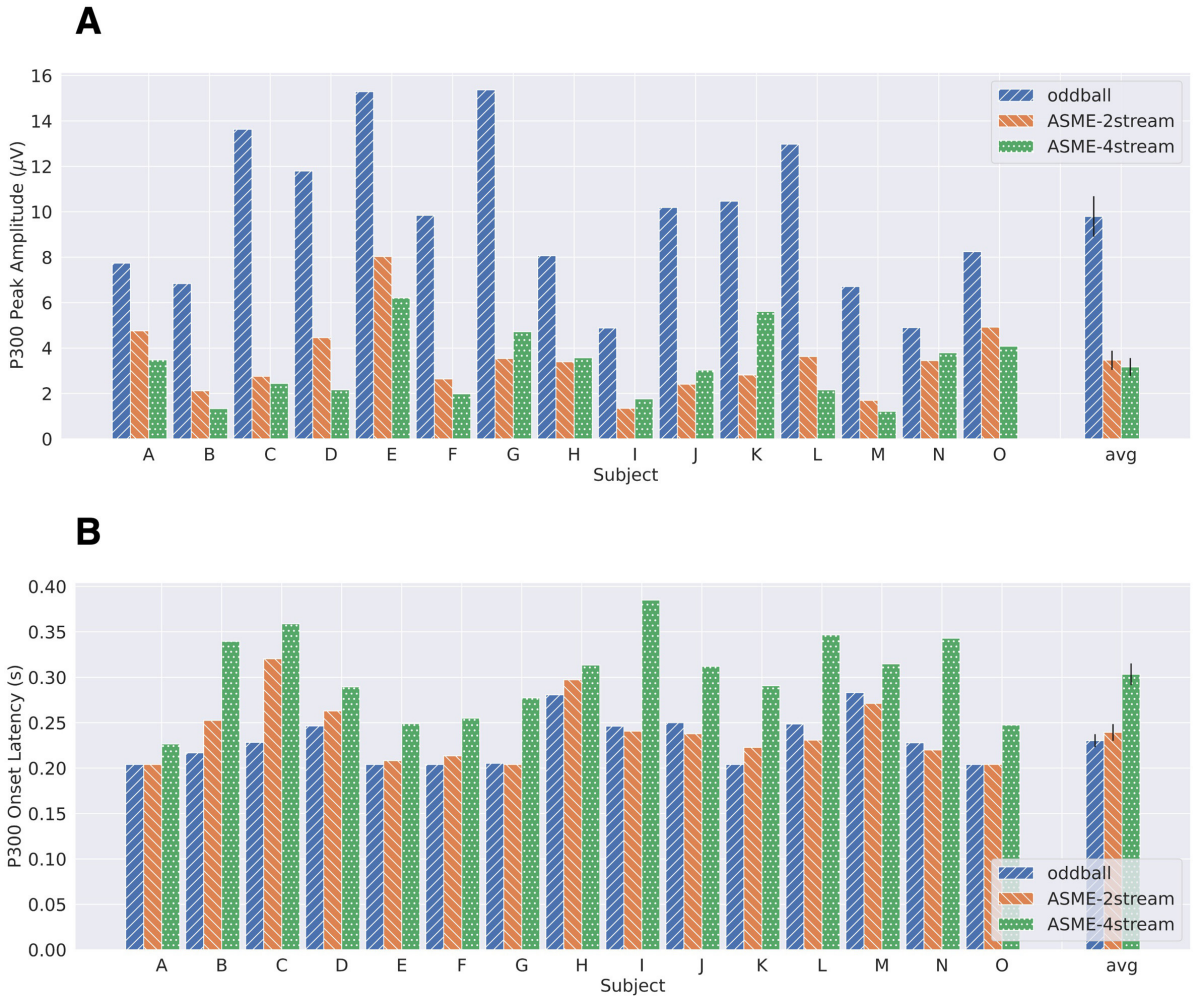
The P300 peak amplitudes **(A)** and onset latencies **(B)** for each subject obtained by the bootstrap procedure. Each error bar shown in an averaged bar plot is the standard error mean.

Figure 6A shows the P300 peak amplitude of three paradigms, i.e., the ASME-2stream, ASME-4stream, and oddball paradigms, obtained using the bootstrap procedure. The number of bootstrap samples for which no significant difference was observed in the analysis described in the Section 2.6 can be found in Supplementary Table S1. The average peak amplitudes from all subjects were 9.80μ*V* (oddball), 3.47μ*V* (ASME-2stream), and 3.17μ*V* (ASME-4stream). There was no significant difference between the ASME-2stream and ASME-4stream groups (*p* = 0.25, two-sided Wilcoxon signed-rank test). Figure 6B shows the P300 onset latency in the three paradigms. The average latencies from all subjects were 0.23 s (oddball), 0.30 s (ASME-4stream), and 0.24 s (ASME-2stream), and the latencies were significantly greater in the ASME-2stream paradigm than in the ASME-4stream paradigm (*p* = 0.61 × 10^−4^, two-sided Wilcoxon signed-rank test).

### 3.2 Classification

The average binary classification scores for all subjects were 0.87 (ASME-2stream) and 0.82 (ASME-4stream). The score in the ASME-2stream was significantly greater (*p* = 0.67 × 10^−3^, two-sided Wilcoxon signed-rank test). The highest scores were 0.96 (ASME-2stream) and 0.94 (ASME-4 stream).

**Figure 7:**
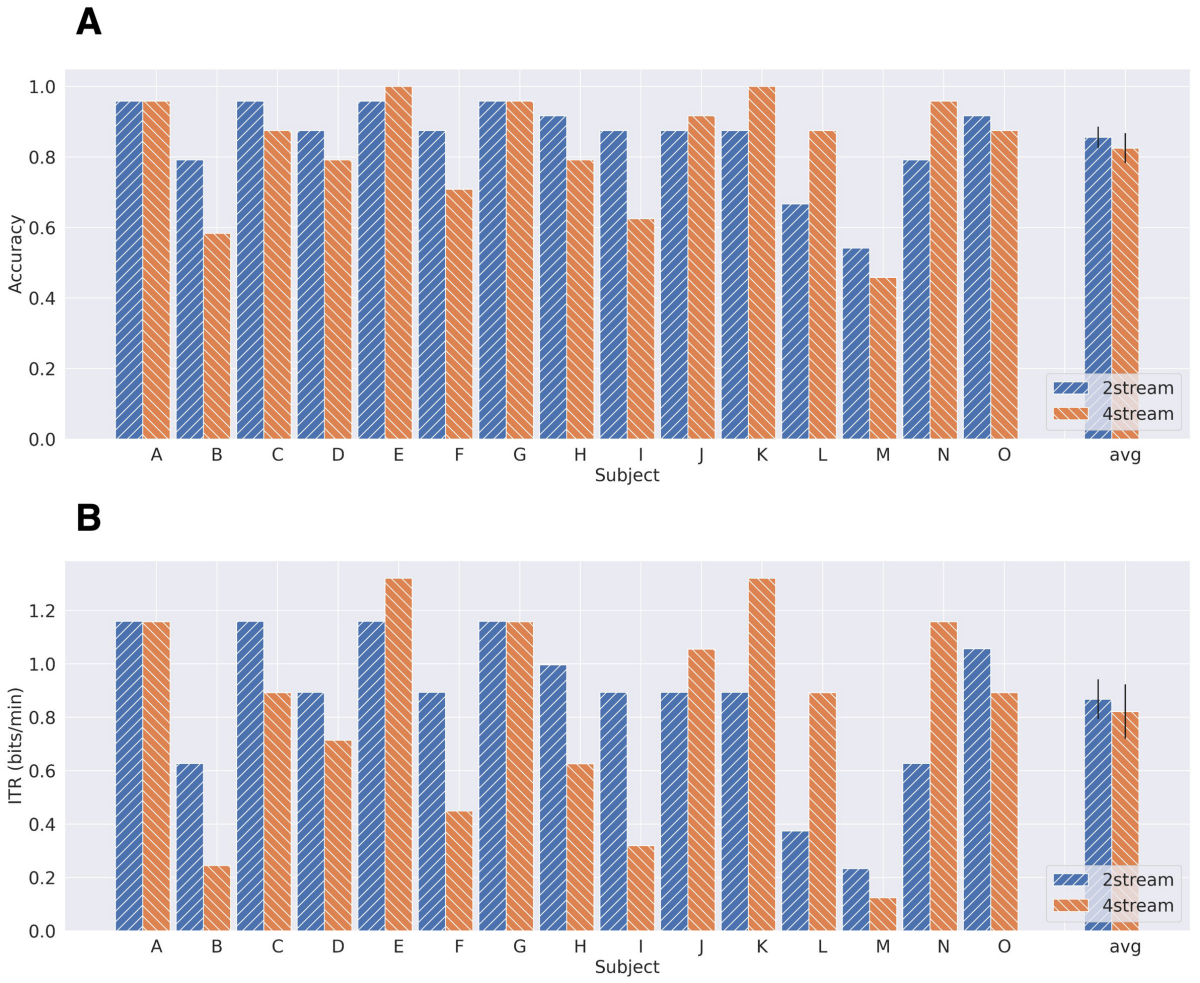
The accuracy **(A)** and ITR **(B)** for each subject obtained by the BCI simulation.

Figure 7 shows the accuracy scores and ITR from the BCI simulation results. The average accuracies were 0.86 (ASME-2stream) and 0.83 (ASME-4stream). However, this difference was not significant (*p* = 0.40, two-sided Wilcoxon signed-rank test). The accuracies for both ASME-2stream and ASME-4stream were higher than statistically significant classification accuracy (0.42).

The maximum accuracies were 0.96 (ASME-2stream) and 1.0 (ASME-4stream). The ITRs were 0.87 (ASME-2stream) and 0.82 (ASME-4 stream), which were not significant (*p* = 0.49, two-sided Wilcoxon signed-rank test). The maximum ITRs were 1.16 (ASME-2stream) and 1.32 (ASME-4stream).

### 3.3 Workload

**Figure 8:**
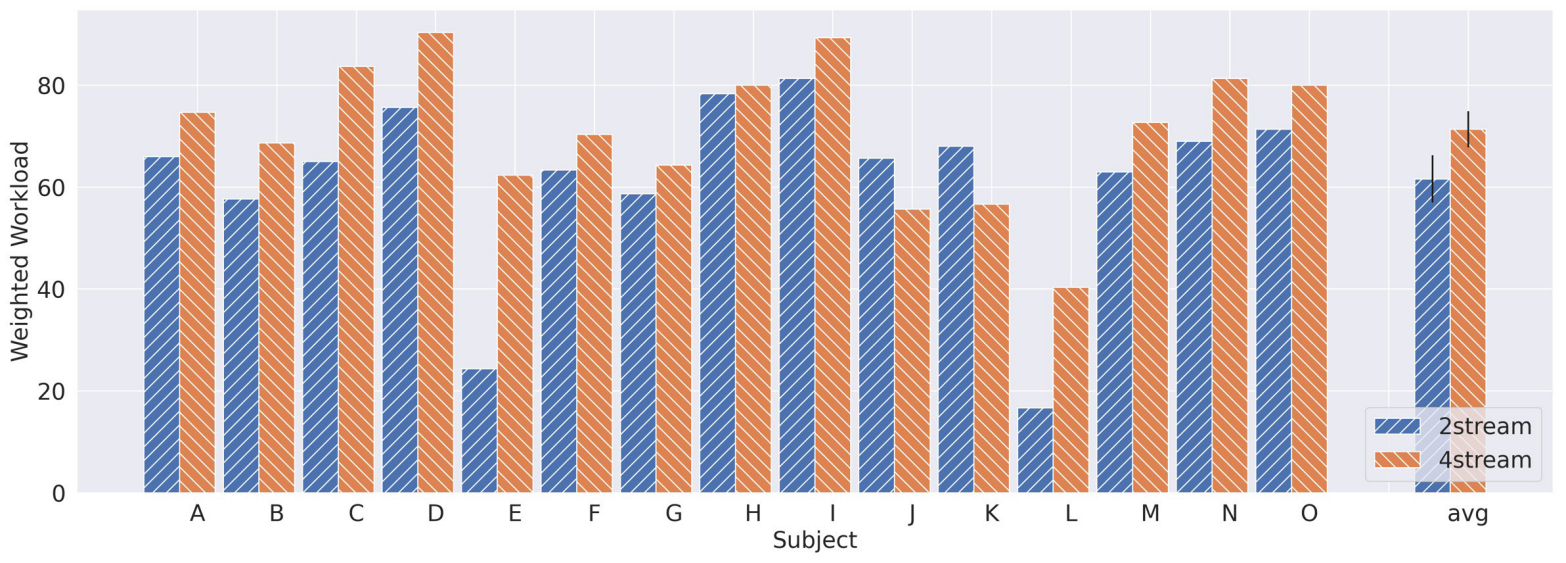
The WWL for each subject was obtained via the NASA-TLX questionnaire.

Figure 8 shows the weighted workload (WWL) obtained by the NASA-TLX questionnaire. The average WLLs of all the subjects were 61.6 (ASME-2stream) and 71.4 (ASME-4 stram), and these differences were significant (*p* = 0.015, two-sided Wilcoxon signed-rank test). This means that the users' subjective workload was significantly greater in the ASME-4stream paradigm than in the ASME-2stream paradigm. Moreover, negative correlations existed between the classification scores and the *performance* index of NASA-TLX (see Table 3). Note that the *performance* index of the NASA-TLX is on the axis, in which a higher value indicates a lower subjective rating of performance. Among the fifteen subjects, the WWL was lower in the ASME-4stream for two subjects. For these two users, the accuracy of the ASME-4stream BCI simulation was also greater than that of the ASME-2stream BCI simulation.

**Table 3:** The correlations between the NASA-TLX performance index and classification performance.

**Paradigm**	**Classification**	**Correlation**	***p*-value**
ASME-4stream	BCI simulation	–0.52	0.046
	Binary classification	–0.48	0.073
ASME-2stream	BCI simulation	–0.64	0.010
	Binary classification	–0.54	0.039

## 4 Discussion

### 4.1 Feasibility of the four-class ASME paradigm

The results of the ASME-4stream paradigm show that it is possible to perceive the presented sequence as four segregated streams and to focus on the target stimuli in the target stream. In addition, the results of the ASME-2stream paradigm show that it is possible to focus on the segregated stream out of two streams and to focus on the target stimulus out of two deviant stimuli. In both paradigms, the accuracy of the BCI simulation (over 0.8 against theoretical chance level = 0.25 and threshold for statistically significant classification accuracy *P*_*th*_ = 0.42) was high enough for the BCI applications. The ITR in both paradigms was less than 1.0 bits/min and lower than that in other proposed methods (Hill et al., 2004; Furdea et al., 2009; Schreuder et al., 2010; Höhne et al., 2011); however, the trial length was long (90 s) in this study, and it can be further improved by optimizing the trial length by applying dynamic stopping methods (Verschore et al., 2012; Schreuder et al., 2013) or other sophisticated methods. It can be concluded that a four-class BCI system can be used; however, only offline analysis was conducted in this study. Hence, online implementation and evaluation are needed for future work.

### 4.2 Comparison of two different four-class ASME approaches

ERP analysis with the bootstrapping procedure showed that the average P300 peak amplitude and its onset latency were greater and shorter, respectively, in the ASME-2stream paradigm. Figure 5 shows that the P300 peak amplitude readout was greater in the ASME-4stream paradigm compared to that in the ASME-2stream paradigm. However, the results from P300 peak amplitude estimation obtained by the bootstrap procedure (Section 2.6) showed larger peak amplitude in ASME-2stream (3.47μ*V* vs. 3.17μ*V*, see Section 3.1). The grand average ERP response waveform in Figure 5 simply shows the average waveform from each subject per EEG channels Cz and Fz. In contrast, with the bootstrap procedure, the peak amplitude of the spatiotemporal feature can be assessed, and it may more accurately reflect the characteristics of ERP peaks. Thus, it can be assumed that the P300 peak amplitude was greater in the ASME-2stream paradigm. Comparing the amplitude and onset latency from the ASME paradigm with those from the simple auditory oddball paradigm, the P300 amplitudes in the oddball paradigm were much larger than those in the two ASME paradigms. However, the P300 onset latency in the ASME-2stream paradigm was on the same level as that in the oddball paradigm, where the P300 latency in the ASME-4stream was significantly greater. According to previous studies (Ghani et al., 2020), the P300 latency was consistently prolonged as task difficulty increased; however, the P300 amplitude was not consistent. Some reported that the P300 increased with increased task difficulty (Combs and Polich, 2006; Muller-Gass and Schröger, 2007); others reported the opposite (Dyke et al., 2015; Horat et al., 2016; Causse et al., 2015; Frank et al., 2012). Hillyard et al. hypothesized that P300 was maximized when “resolution of uncertainty” or “delivery of information” was maximized (Hillyard et al., 1971), which means that tasks that were too clear or too challenging decreased P300 amplitudes; however, the optimal task difficulty maximized the P300 amplitudes. Aggregating these results and findings, it can be concluded that the task difficulty of the ASME-2stream paradigm is lower and more appropriate than that of the ASME-4stream paradigm for eliciting larger P300 amplitudes.

The peak N200 responses were greater in the ASME-2stream paradigm, as shown in Figure 5. However, the average response was broad in the ASME-4stream, suggesting that the latency of the N200 varied across subjects and trials. It was implied that the N200 contributed to the classification due to its large signed-*r*^2^ value.

Frontal-central dominant N700 was also observed in both paradigms; however, the difference between target and nontarget individuals was detected only in the ASME-4stream paradigm. Bender et al. (2010) reported that the amplitude of N700 was enhanced by active short-term memory maintenance compared to attention to current perceptions or passive stimulation. This indicates that more short-term memory maintenance tasks are required in the ASME-4stream paradigm than in the ASME-2stream paradigm. In the ASME-4stream paradigm, the signed-*r*^2^ value for N700 was large, and this component may have contributed to the classification.

The average binary classification and BCI simulation results were better in the ASME-2stream paradigm; however, statistical difference was only observed in the binary classification results. Although the performance is considered comparable for both paradigms, the highest accuracy in this study (accuracy = 1.0 for BCI simulation) was achieved in the ASME-4stream for two subjects, and it was indicated that the ASME-4stream is more suitable for some users to achieve the best performance.

The NASA-TLX results revealed that the subjects' subjective workload was significantly greater in the ASME-4stream paradigm. This suggests that increasing the number of streams increases the user's workload. The classification score and subject's subjective rating of *performance* were also correlated. These results showed that subjective ratings from NASA-TLX can be reflected in quantitative electrophysiological signals and prove the validity of its use in measuring the workload in BCIs. Additionally, this index could indicate which paradigm is the best for the user.

In summary, it can be concluded that the performance is at the same level for both paradigms; however, the user's workload is lower in the ASME-2stream paradigm. Additionally, considering that the best performance was achieved in the ASME-4stream paradigm, using the ASME-4stream paradigm for some users may be adequate to achieve the best performance.

### 4.3 Conclusion

In this study, it was shown that both paradigms, ASME-2stream and ASME-4stream, can be used as BCI systems with high accuracy. From the results of the ASME-4stream, it was shown that focusing on a single stream out of four segregated streams can be possible. The results of the ASME-2stream showed that the ASME paradigm involving multiple deviant stimuli in a single stream may be possible. The average performance across subjects was slightly better in the ASME-2stream paradigm (not significant). According to the WWL of the NASA-TLX, the user's workload is lower in the ASME-2stream, and usability is superior in this paradigm. It was shown that the subjects could carry out the task confidently, and the task difficulty was optimal in the ASME-2stream paradigm. However, it was also suggested that determining which paradigm is the best for the subject is encouraged since some subjects achieved greater performance in the ASME-4stream paradigm. It was also shown that sequences with multiple target stimuli in a single stream can be extended to multiple classes with appropriate task difficulty compared to sequences with a single target stimulus in a single stream in the ASME paradigm. These findings expand the possibility of a multiclass extension of the ASME BCI, providing users with choices of practical auditory BCIs.

## Data Availability

All relevant data are publicly available from the Harvard Dataverse repository (https://doi.org/10.7910/DVN/1UJDV6).
