# Supplementary material for: Four-class ASME BCI: investigation of the feasibility and comparison of two strategies for multiclassing
Source: Front Hum Neurosci. 2024 Nov 26;18:1461960. doi: 10.3389/fnhum.2024.1461960 (PMC11628488; doi:10.3389/fnhum.2024.1461960)
Supplement: Supplementary file 1 [file Data_Sheet_1.pdf]

Supplementary Material

Table S1. The number of bootstrap samples for which no significant difference was observed in the analysis described in the section 2.6.2.

Subject	ASME-2stream	ASME-4stream	Oddball
A	0	0	0
B	0	0	0
C	0	0	0
D	0	0	0
E	0	0	0
F	0	0	0
G	0	0	0
H	0	0	0
I	48	2	0
J	0	0	0
K	0	0	0
L	0	0	0
M	0	0	0
N	0	0	0
O	0	0	0
